# Supplementary material for: Characteristics of the mitochondrial and cellular uptake of MPP+, as probed by the fluorescent mimic, 4'I-MPP+
Source: PLoS One. 2018 Aug 23;13(8):e0197946. doi: 10.1371/journal.pone.0197946 (PMC6107127; doi:10.1371/journal.pone.0197946)

S1 Fig. Standard curves for RP-HPLC-UV quantification of MPP^+ and $4'I\text{-}MPP^+$. The standard curves were constructed from the authentic samples of MPP^+ and $4'I\text{-}MPP^+$. HPLC separation conditions: solvent 48 % buffer containing 20 mM Na_3PO_4, 20 mM CH_3CO_2Na, 30 mM triethylamine, 1.7 mM 1-octanesulfonic acid sodium salt, pH 7.0, and 52% CH_3CN; flow rate 0.8 mL/min. MPP^+ and $4I'\text{-}APP^+$ were detected by UV-Vis at 290 and 310 nm, respectively.

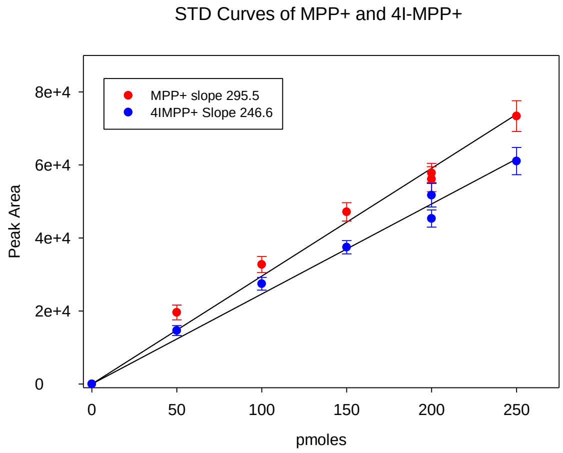

Supplement: S1 Fig — (PDF) [file pone.0197946.s001.pdf]
