# Supplementary material for: Characteristics of the mitochondrial and cellular uptake of MPP+, as probed by the fluorescent mimic, 4'I-MPP+
Source: PLoS One. 2018 Aug 23;13(8):e0197946. doi: 10.1371/journal.pone.0197946 (PMC6107127; doi:10.1371/journal.pone.0197946)

S2 Fig. HepG2 toxicity of MPP^+ and 4'I-MPP^+ at higher concentrations. The toxicities were determine as detailed in Methods. The toxicity of 4'I-MPP^+ could not be accurately determined the concentrations above 200 μM due to the low solubility of the toxin in the incubations media. However, rough estimate shows some cell toxicity above 250 μM concentrations (data not shown).

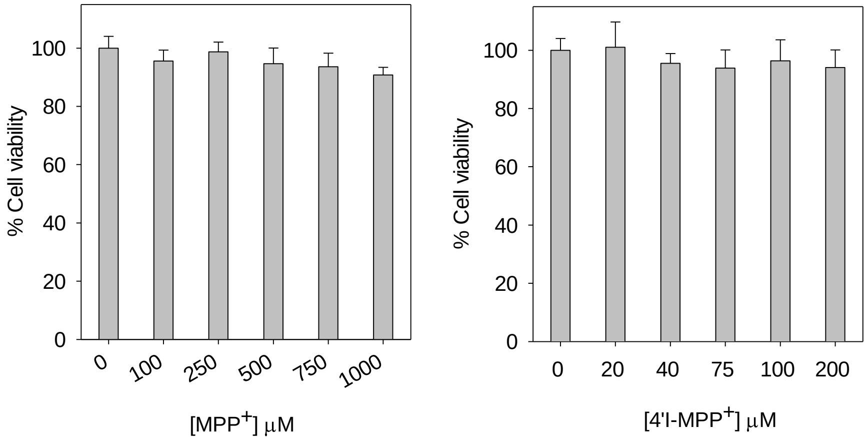

Supplement: S2 Fig — (PDF) [file pone.0197946.s002.pdf]
