# Supplementary material for: Characteristics of the mitochondrial and cellular uptake of MPP+, as probed by the fluorescent mimic, 4'I-MPP+
Source: PLoS One. 2018 Aug 23;13(8):e0197946. doi: 10.1371/journal.pone.0197946 (PMC6107127; doi:10.1371/journal.pone.0197946)

S3 Fig. Standardization of the DCFH-DA based ROS assay in MN9D cells using 1.0 mM H_2O_2. MN9D cells grown in glass bottomed plates were incubated with 10 μM DCFH-DA in KRB-HEPES for 45 min at room temperature. DCFH-DA-loaded cells were washed with KRB-HEPES, 1.0 mL of KRB-HEPES added and mounted on the stage of Nikon ECLIPSE Ti-S inverted fluorescence microscope fitted with a 40X objective. 15-20 ROIs with uniform fluorescence (Ex/Em 488/524 nm) selected cells were imaged in real time at using at 1 min time intervals for 5 min. After baseline was established (5 min) 1.0 mL of freshly diluted 2.0 mM H_2O_2 was added (final concentration 1.0 mM) and the fluorescence measurements were continued for additional 20 min. The averages of background corrected, control subtracted ROI fluorescence intensities were used to determine the intracellular average relative DCF fluorescence change ($\Delta F/F_0$). (A**)** The time course of the generation of ROS in MN9D cells flowing the 1.0 mM H_2O_2 treatment as monitored in real time by measuring increased; (B) DFC fluorescence images of H_2O_2 treated MN9D cells after 12 min; (C) same images of control cells after 12 min.

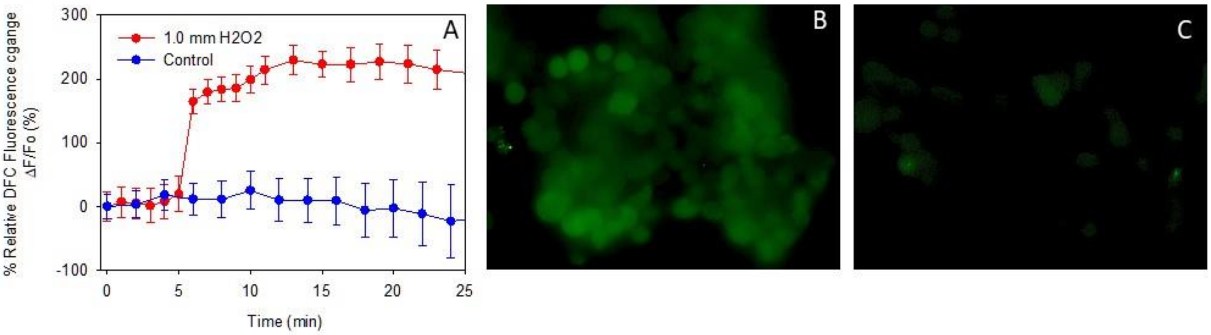

Supplement: S3 Fig — (PDF) [file pone.0197946.s003.pdf]
