# Supplementary material for: Characteristics of the mitochondrial and cellular uptake of MPP+, as probed by the fluorescent mimic, 4'I-MPP+
Source: PLoS One. 2018 Aug 23;13(8):e0197946. doi: 10.1371/journal.pone.0197946 (PMC6107127; doi:10.1371/journal.pone.0197946)

S4 Fig. Effects of Benzamil, Verapamil, FFA, and ruthenium red on the MN9D mitochondrial uptake of 4'I-MPP$^+$. MN9D mitochondrial suspensions were initially incubated with a 50 μM concentration of the desired agent (10 μM Ruthenium red) in KCl buffer for 10 min at 37 °C and then incubated with 200 μM 4'I-MPP$^+$ for 45 min at 37 °C. After the incubations, the intracellular 4'I-MPP$^+$ contents were quantified by RP-HPLC-UV as described in Material and methods. The data are represented as mean ± S.D (n=3).

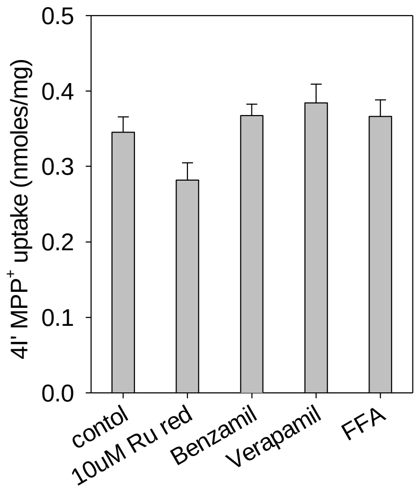

Supplement: S4 Fig — (PDF) [file pone.0197946.s004.pdf]
